# A Case Study of Enhancing the Data Science Capacity of an RCMI Program at a Historically Black Medical College

**DOI:** 10.3390/ijerph20064775

**Published:** 2023-03-08

**Authors:** Qingguo Wang, Vibhuti Gupta, Aize Cao, Ashutosh Singhal, Todd Gary, Samuel E. Adunyah

**Affiliations:** 1 Department of Computer Science & Data Science, School of Applied Computational Sciences, Meharry Medical College, Nashville, TN 37208, USA; 2 Department of Biomedical Data Science, School of Applied Computational Sciences, Meharry Medical College, Nashville, TN 37208, USA; 3 Department of Biochemistry, Cancer Biology, Neurosciences and Pharmacology, Meharry Medical College, Nashville, TN 37208, USA

**Keywords:** data science, RCMI, HBCU, health disparities, diversity

## Abstract

As data grows exponentially across diverse fields, the ability to effectively leverage big data has become increasingly crucial. In the field of data science, however, minority groups, including African Americans, are significantly underrepresented. With the strategic role of minority-serving institutions to enhance diversity in the data science workforce and apply data science to health disparities, the National Institute for Minority Health Disparities (NIMHD) provided funding in September 2021 to six Research Centers in Minority Institutions (RCMI) to improve their data science capacity and foster collaborations with data scientists. Meharry Medical College (MMC), a historically Black College/University (HBCU), was among the six awardees. This paper summarizes the NIMHD-funded efforts at MMC, which include offering mini-grants to collaborative research groups, surveys to understand the needs of the community to guide project implementation, and data science training to enhance the data analytics skills of the RCMI investigators, staff, medical residents, and graduate students. This study is innovative as it addressed the urgent need to enhance the data science capacity of the RCMI program at MMC, build a diverse data science workforce, and develop collaborations between the RCMI and MMC’s newly established School of Applied Computational Science. This paper presents the progress of this NIMHD-funded project, which clearly shows its positive impact on the local community.

## 1. Introduction

With an unprecedented amount of data produced in business, sciences, medicine, social media, and healthcare systems, data analyst skills are becoming increasingly crucial for leveraging big data to gain a competitive edge, accelerate scientific discovery, and advance public health [1]. Accordingly, data science has undergone the fastest growth in the past decade. Its widespread applications have not only transformed industries and the economy but also contributed significantly to scientific discoveries [1,2,3,4]. In particular, the increasing use of data infrastructure, health informatics, and new analytic methods (such as deep learning [5,6,7]) has led to significant benefits to health and health care: cost-effective drug discovery, improved patient outcomes and delivery of patient care, etc.

In computing and information disciplines, African Americans (AA) are among many minority groups who are underrepresented [8,9]. This paucity is especially visible in academic admission, employment sectors and industry [8,9]. Meharry Medical College (MMC), as a Historically Black College/University (HBCU), is the first medical school for AA in the South. Dedicated to educating healthcare professionals and biomedical scientists, it has trained over 40% of America’s AA dentists and produced 8% of the nation’s AA physicians, the majority of whom work in underserved rural or urban communities. Prior to 2021, MMC had only three schools: the School of Medicine, the School of Dentistry, and the School of Graduate Studies. To bridge the representation gaps of AA in computing and to advance health disparity research with data science, in February 2021, MMC established a new school, the School of Applied Computational Sciences (SACS), to provide academic training in data science and computing-related research on a variety of areas including medical, social, and environmental issues that impact the health of minority and underserved populations.

The Research Centers in Minority Institutions (RCMI) Program at MMC (in Health Disparities Research, RHDR@MMC) is a long-term National Institute for Minority Health Disparities (NIMHD)—a funded endeavor that enables high-quality basic, behavioral, and clinical research to eliminate health disparities [10,11,12]. The corresponding U54 award U54MD007586 supports health disparities research in a variety of diseases, such as cardiovascular diseases, cancer, diabetes, HIV/AIDS, and neurological diseases that disproportionally affect the non-Hispanic Black population in the US [10,11,12]. To enhance the research capacity of MMC’s RCMI program, incorporate data science into the health disparities research, and additionally build a diverse data science workforce, a partnership between the two programs at MMC, i.e., RCMI and SACS, is urgently needed.

**Figure 1:**
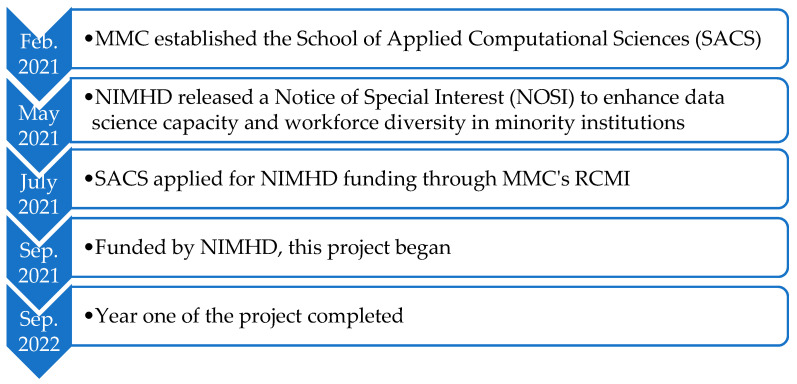
Roadmap of this NIMHD-funded project.

In May 2021, the NIMHD released a Notice of Special Interest (NOSI) entitled “Administrative Supplements to Enhance Data Science Capacity at NIMHD-Funded Research Centers in Minority Institutions (RCMI)”. This NOSI was designed to enhance the data science capacity at Research Centers in Minority Institutions (RCMI) and foster collaborations between RCMI researchers and data scientists. MMC is among the six institutions that applied for and received this award [13] (see Figure 1 for the project roadmap). This NIMHD funding to MMC for this project was awarded to SACS (Award Number U54MD007586-35S5) and administrated through MMC’s RCMI Admin Core. This paper presents the progress and accomplishments of this exciting project for the enhancement of RCMI’s productivity and data science workforce diversity at MMC in the hope of providing guidance and insights to other similar programs interested in replicating or adapting our experience to enhance their institutional data science capacity.

## 2. Materials and Methods

The specific aims of this project were to: (1) Develop authentic and sustainable collaborations between Meharry’s RCMI researchers and data scientists within Meharry’s new School of Applied Computational Sciences; (2) Enhance Meharry’s RCMI data science capacity by providing training to the community; and (3) Assess the learning and data analytics skills of the RCMI investigators, post-docs, medical residents, staff, and/or graduate students and research capacity enhancement of the RCMI program. All our activities for the implementation of these aims in year one of the project, which began in September 2021 and ended in September 2022, were conducted virtually (mostly on Zoom or Team) due to the COVID-19 pandemic.

Many of our project activities, including meetings, seminars, workshops, surveys, and training, were open to the entire Meharry community, even though our funding was focused on the investigators, post-docs, medical residents, staff, and/or graduate students in MMC’s RCMI program. Attendance in our meetings, seminars, workshop, surveys, and training was not mandatory. In addition, the computing needs of the RHDR@MMC program and the effects of data science training were assessed by collecting data from both course participants and instructors. Some of our surveys were disseminated to the entire MMC campus. The data captured were summarized and reviewed by the PI and our data science task force, which had access to both raw data and summarized results.

All our surveys were web-based due to the COVID-19 pandemic, although in general, web surveys generate lower response rates than paper versions (see Appendix A.1 for a web questionnaire administered to RCMI researchers, postdoctoral fellows, residents, staff, and graduate students engaged in our data science training). On the quantitative data collected, descriptive analyses, including percentages and frequencies, were performed. For unstructured narrative text, word clouds were used to identify themes, i.e., the most frequent terms in the text. The word clouds created, however, were not included due to concerns with the small sample size.

## 3. Results

To support the aforementioned specific aims of the project, our activities included (1) creating a joint data science task force of three RMCI investigators and three SACS data science faculty, (2) developing small collaborative research groups that consist of RCMI investigators and data science faculty to work together and generate new ideas, (3) develop and conduct surveys of the RCMI investigators, post-docs, and students, to determine their needs and the areas of data science capacity to enhance in the RCMI program, (4) provide educational mini-grants to RCMI investigators, post-docs, staff, and/or graduate students to cover the costs for taking data science courses; (5) organize a data science summer workshop, etc. Below is a summary of our progress in achieving these project objectives.

### 3.1. Collaborations Established between RCMI Researchers and Data Scientists

When this project began, we faced two unique challenges. First, the faculty in the new School of Applied Computational Sciences did not know RCMI investigators, not to mention collaborations on research and student training. Second, the majority of RCMI investigators, fellows, residents, and medical students at MMC had limited knowledge of data science, and this prevented them from knowing explicitly what data science tools/methods they might need.

To overcome these challenges, a joint Data Science Taskforce was created that consists of three RCMI Core Directors and three SACS data science faculty. This group of diverse and accomplished faculty members from the two programs was proven crucial for the success of this project. The Taskforce discussed the mechanisms to foster collaborations between the RCMI and SACS. With SACS being newly established and its faculty new to MMC, we decided to start by organizing an RCMI Data Science Seminar series every week. Each seminar contained two presenters, an RCMI researcher and a SACS faculty, with the goal of expanding the breadth of discussion topics while promoting joint collaborations between researchers on both sides.

**Figure 2:**
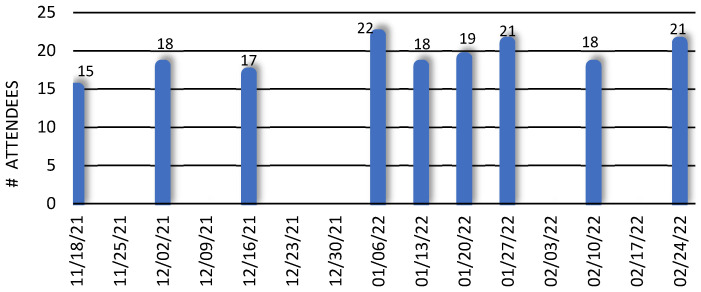
Number of attendees in RCMI Data Science Seminars.

The seminar series began on 18 November 2021 and concluded on 24 February 2022, delivering a total of 18 research presentations. Figure 2 shows the number of attendees at each meeting. The seminar topics included genomic research, deep learning, mobile health, electronic health record (EHR) data analysis, geospatial data analysis, etc. (see Appendix A.2 for specific seminar topics). They not only introduced faculty members and their respective research to one another but also stimulated quality discussions and collaborations among attendees. As a result of these interactions, collaborative research groups were formed between RCMI and SACS to work together on applying data science to health disparities research. In addition, SACS faculty were invited by RCMI investigators to contribute to several NIH R01 proposal submissions and the PI of this project, Dr. Qingguo Wang, was invited to direct a new Genomics Data Core in the RCMI program to provide sequencing-focused services and genomics data science services to the community.

To understand the data science needs of MMC’s RCMI community, a program improvement questionnaire was distributed to each RCMI investigator and SACS faculty who attended the seminar. The data collected was used to assess the needs of the community and to improve the session delivery, structure, and content. From the questionnaire and the seminars, we noticed a gap between the RCMI community and SACS data scientists: the RCMI researchers and students need data, infrastructure, and analytical support to benefit from big data or data-intensive collaborations, whereas the data scientists need domain knowledge in basic research and clinical applications to assist RCMI researchers. To narrow this gap, a half-day summer workshop was organized in August 2022. To address it more effectively, however, a long-term endeavor and continuous funding support are needed.

### 3.2. Mini-Grants to Promote Collaborations between RCMI and SACS to Enhance Health Disparities Research

As a means to foster long-term collaborations between the data scientists and RCMI faculty, this project offered four mini-grants ($15,000 each) for collaborative research activities. In addition to scientific merits, we requested each mini-grant application must meet two requirements: (1) each research proposal must be led by two principal investigators: one RCMI researcher and one SACS faculty; and (2) the proposal should demonstrate the potential to promote health equity or enhance RCMI’s data science capacity. In addition, applicants were highly encouraged to engage minority students in research to train next-generation data scientists. Our hope was that these short-term collaborative projects result in increased (1) RCMI’s data science capacity, (2) external funding, and (3) diversity in the data science workforce.

In total, six groups applied for the four mini-grants, reflecting the level of collaboration developed between the RCMI researchers and data scientists. To make the mini-grant review process fair and transparent, an independent Sub-award Review Organization Committee (SROC) was set up to establish a review process. The SROC made collective decisions on issues from designing the review process and selecting and inviting reviewers to appoint a chair of the review panel. Three external and three internal reviewers were recruited and assigned randomly to the applications. Reviewers scored the proposals based on the established NIH criteria and met them as a review panel (chaired by a senior investigator who is experienced with NIH study sections). Table 1 below illustrates the four applications selected by SROC to receive the mini-grants. Their titles indicate the multiple aspects of health disparity research they proposed to address using data science.

### 3.3. Data Science Capacity Building through Training

**Figure 3:**
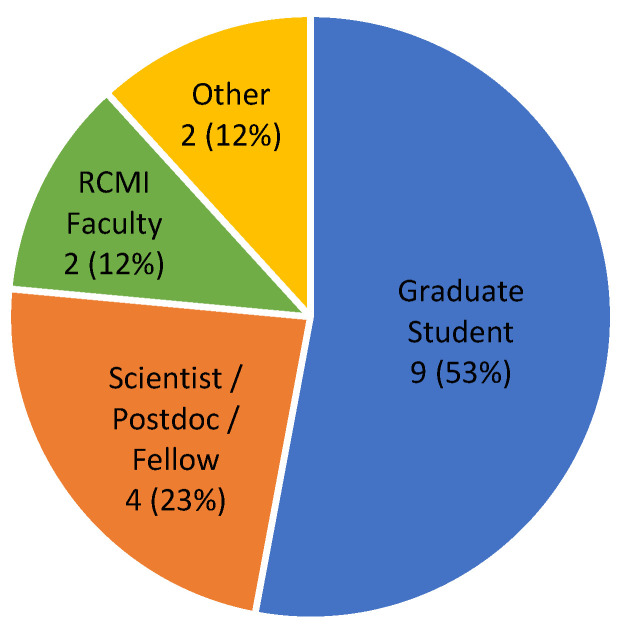
Diversity of applicants for our data science training.

To enhance the data science capacity of the RHDR@MMC program, in Spring 2022, we began providing data science training to the RCMI community. Initially, we planned to offer 12 educational mini-grants to pay for the expenses of the RCMI investigators, scientists, residents, staff, and graduate students for taking SACS data science courses. After announcing the training opportunity, 15 RCMI researchers, students, and staff applied for the mini-grants within ten days (the application site was taken offline ten days after the announcement). As shown in Figure 3, in total, 17 applied for our mini-grants: 9 (53%) of them were graduate students, 4 (23%) were scientists/postdoctoral fellows, and 2 (12%) were RCMI faculty. With respect to the schools, nine applicants were from the School of Graduate Studies, two were from the School of Dentistry, and 5 were from the School of Medicine. These figures show the strong interest in data science within the broad and diverse Meharry community. The questionnaire administered to RCMI researchers, postdoctoral fellows, residents, staff, and graduate students engaged in data science training is demonstrated in Appendix A.1.

**Figure 4:**
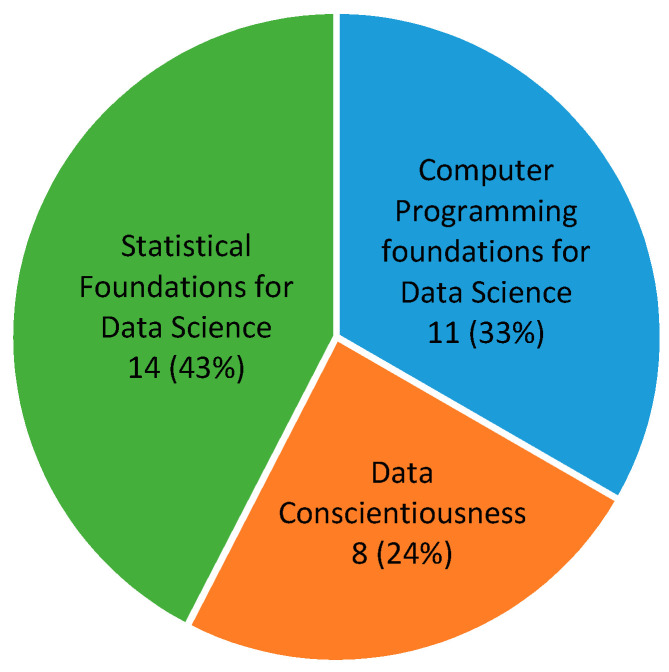
Number of requests for three data science courses offered by SACS.

The SACS’s data science programs began in August 2021, and its first courses were part of the data science training in this program. These included three beginner-level courses: (1) Computer Programming Foundations for Data Science, (2) Data Conscientiousness, and (3) Statistical Foundations for Data Science, which reflect three important data analyst skills for students. Figure 4 shows the number of requests for each of the three training courses (an applicant was allowed to take more than one course). Fourteen trainees (43%) applied for a statistical foundation course, indicating a great interest in statistical modeling in Meharry’s RCMI program. Some students also expressed great interest in more advanced topics, such as Artificial Intelligence (AI) and Machine Learning (ML). Though unable to accommodate their need at the time, we invited two professors in the field to cover AI/ML in our summer workshop. We plan to offer more AI/ML-related training in the future.

To avoid conflict with the busy schedule of the RCMI investigators, staff, medical residents, staff, and graduate students, data science training was offered twice per week in the evening after 5:30 p.m. When attempting to admit students into the courses, however, we found that around half of them who were funded by mini-grants eventually did not make it despite their interest in data science. To figure out the barriers preventing MMC students from participating in data science training, a survey was sent to the entire campus. Twenty-nine students responded. Twenty-five (86.2%) of them showed either interest or strong interest in Data Science, reaffirming our earlier observation. When asked, “How does your current school schedule affect your thoughts on taking data science courses?” sixteen respondents (55.2%) said their school schedules interfere with data science training. Along with school schedules, affordability and course delivery methods also affect their decisions. 

It is worth noting that the data collected from our training program have been combined with those from five other NIMHD-funded minority-serving institutions for a joint publication [13], which as a cross-sectional survey, complements this work regarding curricular design, topics covered, and delivery methods.

### 3.4. Learning Assessment

**Figure 5:**
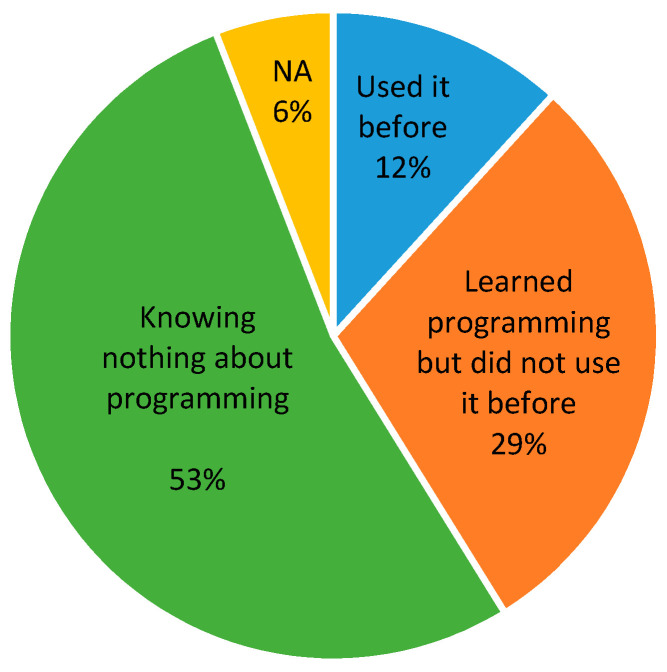
Programming skills of the RCMI researchers, postdoctoral fellows, residents, staff, and graduate students engaged in data science training.

The data science trainees recruited in this program were composed of more than 50% African Americans and 60% female. Prior to the start of the training, their computing knowledge and skills were evaluated to guide course delivery (see Appendix A.1 for survey questions). Figure 5 shows the programming skill levels of the trainees. It shows only 12% percent of them had used a programming language before, and 53% did not know anything about it.

This survey alerted us that these participants were entering the data science training courses with limited experience in computer programming. In the Fall of 2022, we assessed their learning and found all the trainees successfully completed the data science courses. Despite the overall low levels of programming skills of the RCMI researchers and students (as shown in Figure 5), they performed well in course participation, homework assignments, and course projects. Their overall performance as measured by course grades was also excellent, with none receiving a grade lower than B. This indicates they had acquired data science skills from the training and that the training program was successful for these students. To compare their performance with the regular students in SACS, a Mann–Whitney U test was performed [14]. With a *p*-value of 0.73, the test did not find a statistically significant difference between the grades of the two groups.

## 4. Discussion

This paper presents our NIMHD-awarded study that aimed to enhance the data science capacity of MMC’s RCMI program, develop a diverse data science workforce, and foster collaborations between the RCMI and the newly established School (SACS) at MMC. Even though SACS was new and collaborative relationships between SACS and RCMI did not exist at the beginning of this project, significant progress has been made, as reported in this paper.

Due to the limited nature of this study, the number of students involved in our project was relatively small. The tight project timeline and SACS’s shorthandedness at the time also set a limit on the number of students that could possibly engage due to the intense time and efforts required to work with the students: setting up application (survey) websites from scratch, reviewing and processing mini-grant applications, admitting students in training courses, etc. Although the number of students involved met our goals, efforts are needed to expand the sample size in subsequent studies as we expand our study scope in the future.

It is worth noting that besides sample size, this study has two other limitations: first, with our work focused exclusively on the participants at Meharry Medical College, an HBCU, our findings and lessons learned may not generalize well to other different institutions. Second, although our data science training in year one generated great excitement among attendees, they concentrated on generic methodology without addressing the specific needs of the RCMI investigators, post-docs, staff, medical residents, and/or graduate students. To address this issue, we applied for and received a renewal award to implement a more targeted approach in year two. We anticipate more continuous funding in the future will be needed to help meet the research needs of the RCMI community and build a truly diverse data science workforce.

## 5. Conclusions

Data science as a new field has grown tremendously in the past decade due to its great potential to transform industries, the economy, health care, and scientific discovery. With data analyst skills becoming increasingly crucial for leveraging big data to gain a competitive edge, accelerate scientific discoveries, and advance public health [1,15,16,17,18], the inequitable representation of minority groups in the field, coupled with the lack of resources and infrastructure in minority-serving institutions, has become a major issue in the U.S.

This paper presented our NIMHD-funded project on addressing the growing need for incorporating data science into health disparities research at Meharry Medical College and enhancing workforce diversity in the field of data science. Our results showed that this work fostered collaborations between the RCMI researchers and the data science faculty. Significant progress has also been made in enhancing the overall data science capacity of the RCMI program by offering mini-grants to research groups and training investigators, staff, medical residents, and graduate students in data analyst skills. Because this paper came out of a pilot project with limited funding, we believe that this work provides important information that would be vital for future scaling-up studies. Furthermore, we hope that our experience learned can be replicated by other minority-serving programs that are interested in enhancing their data science capacity and building a diverse workforce at their institutions.

## Figures and Tables

**Table 1:** Collaborative projects selected for mini-grants by our Sub-award Review Organization Committee.

Project Titles	# Students Involved
The role of circRNAs in prostate cancer malignancy and disparities	
2. A retrospective study to examine the correlation between high cholesterol and substance use disorder	1
3. Understanding BRCT–domain containing protein function in cellular signaling using network data science on multi-omics and interaction data.	1
4. Role of S. cristatus in modulation of oral microbiome	

## Data Availability

Part of our data was archived publicly at: https://doi.org/10.7910/DVN/UG4OM7 (accessed on 30 January 2023).

## Appendix A

### Appendix A.1. Questions Administered to RCMI Researchers, Postdoctoral Fellows, Residents, Staff, and Graduate Students Engaged in Data Science Training

**Table:**

Question #	Survey Question	Type of Question
1	Which of the following data science courses do you want to take in Spring 2022? Computer Programming Foundations Data Conscientiousness Statistical Foundations for Data Science	Multiple Choice
2	Please tell us who you are.∙ I am a graduate student. I am a scientist/postdoc/fellow/staff. I am an RCMI faculty/PI/Core Director. None of the above.	Multiple Choice
3	Please tell us which school you are from? School of Medicine School of Dentistry School of Graduate Studies None of the above	Multiple Choice
4	Please tell us if you took the following math courses before: Calculus Algebra Statistics	Multiple Choice
5	Choose one that is closest to your current programming skill level: I use programming language(s) frequently. I learned it before but did not use it much. I did not know anything about it. None of the above.	Multiple Choice
6	Select any of the following topics that you are familiar with: Scripting languages Data Structures Algorithms∙ Databases Discrete Math Operating Systems	Multiple Choice
7	What is your field of research or work that you would like to apply data science?	Text Input
8	Please leave your question or comment below if you have	Text Input

### Appendix A.2. Topics of RCMI Data Science Seminars

**Table:**

**Date**	**Seminar Topic**
18 November 2021	Sequencing Data Analyses and Their Applications to Cancer Research Does elevated cholesterol affect psychostimulant addiction?
2 December 2021	A small molecule inhibitors HIV-1 replication by restoring APOBEC3G antiviral function Evidence Based Health and Race Disparity Studies
16 December 2021	A Comprehensive Analysis of the Effect of Histological Subtypes on the Survival Probability of Kidney Carcinoma Patients: A Hypertabastic Survival Analysis Digital Health Devices Data Stream Analytics for Early Insights into life-threating Diseases
6 January 2022	Meharry Bioinformatics + Proteomics Core Update: BioMedical Informatics Projects and Proteomics Upgrades Protein Sequence analysis using Machine Learning
20 January 2022	Cancer/Ribonucleoprotein granules/ZAR1L Softwarized Network
27 January 2022	Some Applications of Machine Learning in Healthcare Precision Medicine Using Structural Biophysics and Protein Dynamics
10 February 2022	Geospatial Data and Healthcare Research Research on Atherosclerosis
17 February 2022	Oncogene KDM5B in human prostate cancer The cBioPortal for Cancer Genomics
24 February 2022	Averting Neonatal Abstinence Syndrome (NAS) Among Young Women Attending Syringe Services Programs Chronic Neural Stressor Agents within Dysautonomic and Arrhythmic Disorders

Note: The second talks in the last two seminars in February 2022 were given by invited external speakers.
